# Position of Maxillary Lateral Incisor and First Premolar in Impaction of Maxillary Canines: A Controlled Clinical CBCT and 3D Study Model Analysis

**DOI:** 10.3390/dj13110497

**Published:** 2025-10-27

**Authors:** Maja Hočevar, Maja Ovsenik, Aljaž Golež

**Affiliations:** 1 Department of Orthodontics and Dentofacial Orthopaedics, University Medical Centre of Ljubljana, 1000 Ljubljana, Slovenia; 2 Department of Orthodontics and Dentofacial Orthopaedics, Faculty of Medicine, University of Ljubljana, 1000 Ljubljana, Slovenia; 3 Institute of Physiology, Faculty of Medicine, University of Ljubljana, 1000 Ljubljana, Slovenia

**Keywords:** impacted canines, CBCT, adjacent teeth displacement, dental follicle, controlled clinical trial

## Abstract

**Objectives**: This study examined the link between impacted maxillary canines and changes in the position of adjacent lateral incisors (LIs) and first premolars (FPs), as well as opposite canines. It also explored the relationship between the position of impacted canines and the presence of palpable mucosal bulges. **Methods:** The clinical study involved 62 participants (35 females, 27 males; average age: 14.7 years), split equally into impacted canine (n = 31) and control (n = 31) groups. The study included 26 palatally impacted canines, 5 buccally impacted canines, 31 non-impacted contralateral canines, and 62 control canines. Three-dimensional study models assessed LI and FP positions, while CBCT analyzed vertical and horizontal positions of impacted canines and dental follicles. Clinicians evaluated the presence of mucosal bulges, and distance was measured between impacted canines and outer cortical bone radiographically. **Results:** Compared to controls, the LIs next to impacted canines showed significantly more rotation (13°), mesiodistal angulation (11.5°), and buccopalatal angulation. FPs showed increased rotation (10.0°) and mesiodistal angulation (8.7° more) but no change in buccopalatal inclination. Contralateral canines had significantly more rotation (11.3°) than controls. Buccally impacted canines led to greater positional differences in the LIs compared to palatally impacted canines. Follicle thickness had a moderate correlation with FP rotation and mesiodistal angulation but did not relate to LI malposition. Visible mucosal bulges indicated distances of 1.2 mm or less between the canine and cortical bone. **Conclusions:** This study found positional differences in lateral incisors showed altered rotation, angulation, and inclination, while first premolars had increased rotation and angulation. Buccally impacted canines showed more discrepancies for lateral incisors. A mucosal bulge may indicate impacted canine location if the impacted canine is close to the outer bone surface. Follicle thickness affected premolar position but not incisors.

## 1. Introduction

An impacted tooth is defined as a tooth that has failed to erupt into the oral cavity within the expected developmental period but has become obstructed for various reasons and remains embedded in the jawbone [1]. The mean age of eruption for maxillary canines is typically 10.8–11.6 years [2]. A tooth normally erupts when half to three quarters of its final root length has developed [3]. Impacted maxillary canines is a common condition, affecting between 0.8 and 2.8% of the population [4,5]. This makes them the second most frequently impacted teeth after the lower third molars [6,7]. They are more frequently impacted in women than in men, with a ratio of 2:1 [8]. Upper canines can be impacted palatally (85%) or buccally (15%) [9]. Unilateral impaction is more common and occurs in 92% of cases, while bilateral impaction occurs in only 8% of cases [10]. The most common etiological factors for canine impaction include incorrect positioning or shape of the dental germ, a narrow dental arch space, tooth gaps, odontomas, infections, cysts, supernumerary teeth, disorders in the development of the incisors, trauma and genetics [11]. Buccally impacted maxillary canines are more commonly associated with crowding, while the etiology of palatally impacted canines is nowadays explained by two main theories: the ‘Guidance Theory’ states that the position and development of the root of the maxillary lateral incisor has a direct influence on the eruption of the maxillary canine [12,13]. The ‘Genetic Theory’ states that it is a form of genetic variation that may include various genetic dental anomalies in addition to the palatally impacted canine, associated with dental phenotypes such as aplasia of the lateral incisors or second premolars, hypo-plastic lateral incisors, infra-occlusion of the deciduous molars and a deep bite with distal occlusion and retroinclination of the lateral incisors [14,15,16]. Early diagnosis of an impacted canine is very important to prevent possible negative effects such as root resorption of the neighboring teeth, mesial migration of the premolars, space closure, ankylosis of the canine and the development of cysts or recurrent infections [17,18]. Clinical monitoring of the eruption process of the upper canines usually begins at the age of nine. If the canine cannot be palpated or if it is palpated in an unusual location, or if there is asymmetry in the eruption of the canine on the contralateral side of the dental arch for at least six months, there is sufficient reason to suspect eruption disorders, and a panoramic radiograph is indicated [19].

A few studies have investigated the influence of impacted canines on the position of the maxillary lateral incisor and the first premolar [20,21,22,23,24]. In palatally impacted canines, the common findings are mesio-buccal rotation of the lateral incisor, mesial angulation of the lateral incisor, buccal tipping of the lateral incisor root and mesio-buccal rotation of the first premolar. In patients with buccally impacted canines, the typical findings are mesio-buccal rotation and palatal tipping of the lateral incisor root [21].

Previous studies have primarily used CBCT scans to investigate positional abnormalities of the lateral incisor and the first premolar in patients with impacted canines [20,25]. However, to our knowledge, there are no studies that have clinically evaluated the spatial orientation of the lateral incisor and the first premolar using 3D scans or compared and combined these results with the position of the impacted canine relative to the neighbouring teeth on CBCT [26], which provided the basis for the present study. An additional objective of this study was to determine a threshold distance between the impacted canine and the outer cortical bone necessary for a clinically detectable mucosal bulge, which has not been addressed in previous research. Furthermore, the role of dental follicles in canine impaction and the associated positional discrepancies in neighboring teeth have not been yet insufficiently understood. Therefore, this study aimed to investigate the influence of the impacted canine and its follicle on the position of adjacent teeth.

## 2. Materials and Methods

This controlled clinical study was conducted on subjects with a unilaterally impacted maxillary canine during the late mixed to permanent dentition (study group) and on subjects in the late mixed to permanent dentition without an impacted canine (control group). Ethical approval for this study was obtained from the Slovenian Ethical Committee, Ljubljana, Slovenia, No. 0120-83/2025-2711-3, approval date: 25 March 2025.

The study group and control group of subjects were randomly selected from the pool of patients referred to Orthos Institute, Ljubljana, Slovenia and from The University Medical Centre Ljubljana, Slovenia.

The final sample was selected by the following criteria:

**Inclusion criteria:**

Impacted canine group:

- Late mixed dentition or permanent dentition, with or without a retained primary canine;
- Buccal or palatal unilateral impaction of the maxillary canine;
- Optimal intraoral 3D study model and CBCT scan prior to treatment.

Control group:

- Late mixed dentition or permanent dentition;
- Availability of an intraoral 3D study model.

**Exclusion criteria:**

- The patient was undergoing orthodontic treatment prior to the CBCT scan and 3D scan;
- Bilateral impaction of the maxillary canines;
- Aplasia of the maxillary lateral incisor and/or maxillary first premolar;
- Presence of pathology in the maxilla (e.g., cysts, odontomas, other impacted teeth);
- Hypoplastic lateral incisors;
- Congenital craniofacial anomalies or syndromes;
- History of trauma to the face or teeth.

The study included 62 participants (35 females, 27 males; average age: 14.7 years), evenly divided into two groups: 31 participants in the study group and 31 participants in the control group.

Each patient with a unilaterally impacted canine was invited to participate. Control group participants were selected in order to match the impacted canine group according to age, gender and their skeletal maturity according to CVM stage [27].

Based on preliminary data on the prevalence of morphological abnormalities in adjacent teeth associated with impacted canines, we estimated the required sample size to achieve a statistical power of 0.8 and a significance level (α) of 0.05 for an independent two-sample *t*-test. This calculation indicated that at least 35 participants (18 for each group assuming equal distribution between experimental and control group) would be required to detect a statistically significant difference in the occurrence of positional abnormalities in adjacent neighboring teeth of the impacted canine.

The study was based on the analysis of digital 3-D study models using Dolphin 3-D imaging software, version 11.95), (Patterson Dental Holdings, Inc., Chatsworth, CA, USA) and CBCT computed tomography OnDemand3D Dental Viewing Software, version 1.0 (CyberMed, Seoul, Republic of Korea) and Planmeca Romexis 3-D Imaging Software, version 3.1.0.R, (Planmeca group, Helsinki, Finland) created as part of the diagnostic evaluation of participants prior to the start of orthodontic treatment. Measurements were taken on the 3D study models to assess the rotation, mesio-distal angulation (M-D angulation) and buccopalatal inclination (B-P inclination) of the maxillary permanent lateral incisor and first premolar. The CBCT scans were used to evaluate the vertical and horizontal positions of the impacted maxillary canine and its dental follicle in relation to the maxillary lateral incisor and first premolar. The minimum distances between the impacted canine and the neighbouring lateral incisor and first premolar were measured. The study also included the measurement of the size of the dental follicle of the impacted maxillary canine and the minimum distances between the dental follicle and the neighbouring maxillary lateral incisor and the first premolar. In addition, the presence of a buccal or palatal bulge was assessed on 3D digital study models, while the shortest distance between the impacted canine and the outer cortical bone margin was measured on CBCT images.

### 2.1. 3D Study Model Evaluation (Figure 1A–F)

**Figure 1:**
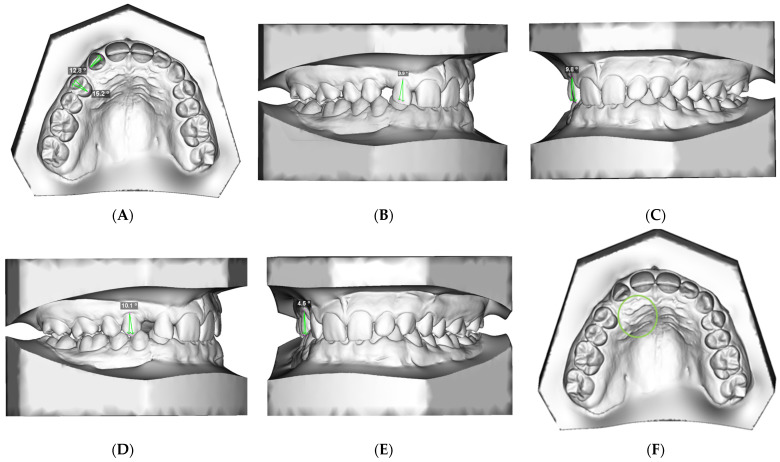
3D study model evaluation: (**A**) rotation of the lateral incisor and first premolar; (**B**) M-D angulation of the lateral incisor; (**C**) B-P inclination of the lateral incisor; (**D**) M-D angulation of the first premolar; (**E**) B-P inclination of the first premolar; (**F**) assessment of presence of the buccal or palatal mucosal bulge of the impacted canine.

**A:** Rotation of the lateral incisor and first premolar: The angle between the line of the occlusal aspect of the crown and the line of the ideal arch form (Figure 1A).

**B:** Mesiodistal (M-D) angulation of the lateral incisor: The angle between the frontal long axis of the lateral incisor and the vertical line parallel to the midline (Figure 1B);

**C:** Buccopalatal (B-P) inclination of the lateral incisor: The angle between the long axis of the lateral incisor and the vertical line parallel to the midline (Figure 1C);

**D:** Mesiodistal (M-D) angulation of the first premolar: The angle between the long axis of the first premolar and the vertical line parallel to the midline (Figure 1D);

**E:** Buccopalatal (B-P) inclination of the first premolar: The angle between the long axis of the first premolar and the vertical line parallel to the midline (Figure 1E);

**F:** Assessment of mucosal bulge: The presence of a buccal or palatal mucosal bulge of the impacted canine (Figure 1F).

### 2.2. CBCT Evaluation (Figure 2, Figure 3 and Figure 4)

**Figure 2:**
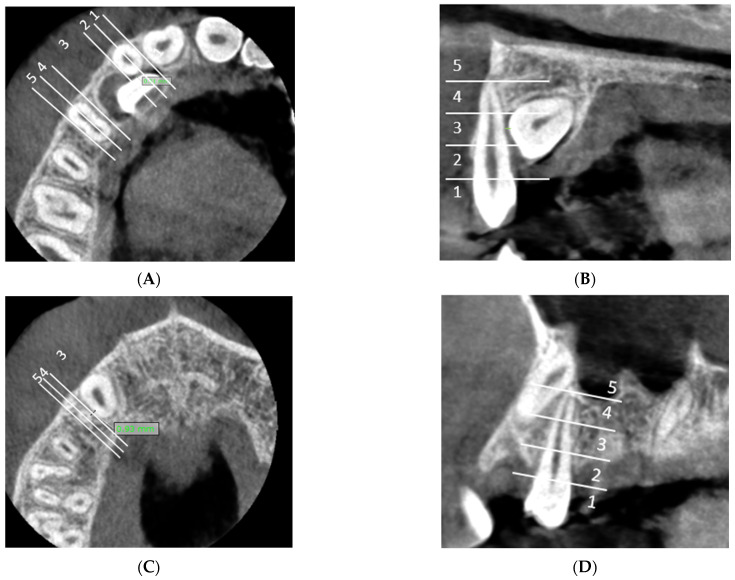
CBCT evaluation: (**A**) horizontal zone of impaction of the maxillary canine in relation to the lateral incisor (axial CBCT slices): the shortest distance between the lateral incisor and the impacted canine is located in horizontal impaction zone 2. (**B**) Vertical zone of impaction of the maxillary canine in relation to the lateral incisor (sagittal slices): the shortest distance between the lateral incisor and the impacted canine and is located in vertical impaction zone 3. (**C**) Horizontal zone of impaction of the maxillary canine in relation to the first premolar (axial CBCT slices): the shortest distance between first premolar and the impacted canine is located in horizontal impaction zone 3. (**D**) Vertical zone of impaction of the maxillary canine in relation to the first premolar (sagittal slices): the shortest distance between the first premolar and the impacted canine is located in vertical impaction zone 5.

**A:** Measurement of the shortest distance between impacted canine and lateral incisor and classification of the shortest distance in horizontal zone of impaction (Figure 2A).

**B:** Classification of the shortest distance within the vertical zone of impaction relative to the lateral incisor (Figure 2B).

**C:** Measurement of the shortest distance between impacted canine and first premolar and classification of the shortest distance in horizontal zone of impaction (Figure 2C).

**D:** Classification of the shortest distance within the vertical zone of impaction relative to the first premolar (Figure 2D).

We divided the horizontal and vertical dimensions into five zones (1–5 zones) (Figure 2).

Horizontal zone of impaction (Figure 2A,C):

- Horizontal zone 1: Mesial half of the maxillary lateral incisor.
- Horizontal zone 2: Distal half of the maxillary lateral incisor.
- Horizontal zone 3: The area between distal point of the lateral incisor and mesial point of the first premolar.
- Horizontal zone 4: Mesial half of the maxillary first premolar.
- Horizontal zone 5: Distal half of the maxillary first premolar.

Vertical zone of impaction (Figure 2B,D):

- Vertical zone 1: The area inferior to the cementoenamel junction of the lateral incisor/first premolar.
- Vertical zone 2: The lower third of the vertical length (measured from the cementoenamel junction to the apex) of lateral incisor/first premolar.
- Vertical zone 3: The middle third of the vertical length (measured from the cementoenamel junction to apex) of lateral incisor/first premolar.
- Vertical zone 4: The upper third of the vertical length (measured from the cementoenamel junction to the apex) of lateral incisor/first premolar.
- Vertical zone 5: The area superior to the apex of the lateral incisor/first premolar.

**Figure 3:**
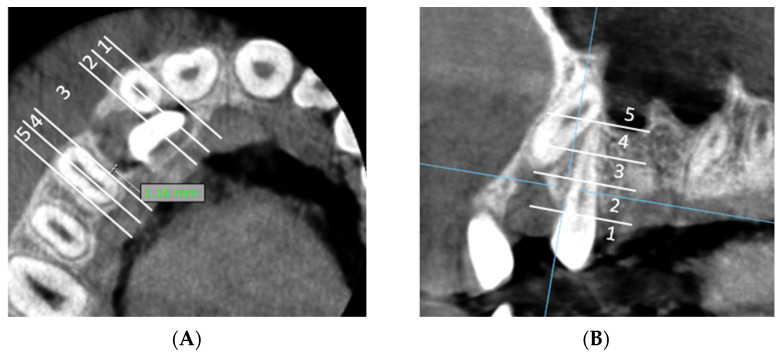
(**A**) The minimal distance between the dental follicle of the impacted canine and the adjacent upper lateral incisor is 0 mm, indicating direct contact between the distal surface of the lateral incisor’s root and the dental follicle, and is classified in horizontal zone 2. The minimal distance between the dental follicle of the impacted canine and the adjacent first premolar is 1.16 mm and is classified in horizontal zone 3. (**B**) The shortest distance between the dental follicle of the impacted canine and first premolar is located in vertical impaction zone 2.

**E:** Measurement of the shortest distance between the dental follicle of the impacted canine and the lateral incisor/first premolar and assessment of horizontal and vertical zone of the canine follicle in relation to the lateral incisor/first premolar (Figure 3A,B).

**Figure 4:**
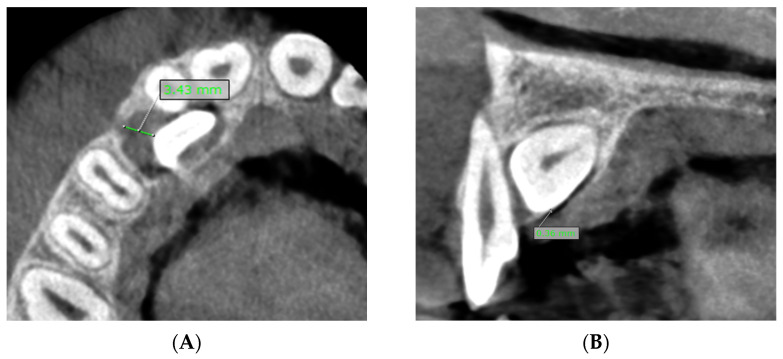
(**A**) Measurement of the maximal follicle size of the impacted maxillary canine. (**B**) Measurement of the shortest distance between the impacted canine/dental follicle and the external cortical bone margin. The minimal distance between the dental follicle and the external cortical bone margin is 0 mm and the minimal distance between the impacted canine and the external cortical bone margin is 0.36 mm.

**F:** Measurement of the maximal follicle size of the impacted maxillary canine (Figure 4A).

**G:** Measurement of the shortest distance between the impacted canine/dental follicle and the external cortical bone margin. (Figure 4B).

## 3. Statistical Analysis

The data were recorded using Microsoft Excel (MS Office, Microsoft corp., Redmond, WA, USA) and analyzed using SigmaPlot (v14.0) (Grafiti LLC., Palo Alto, CA, USA). Statistical significance was set at *p* ≤ 0.05 (95% confidence interval). Each parameter was tested for normality using the Shapiro–Wilk test and non-parametric analysis was used in the case of a non-normal distribution.

Descriptive statistics and one-way frequency tables were used to describe the impacted canine and control groups. Group differences in gender were assessed using the chi-square test, while age differences and skeletal maturity were assessed using the Mann–Whitney rank sum test. Data are presented as mean ± standard deviation (SD) or median (25th–75th percentile) unless otherwise stated. Reliability of continuous measures was calculated using the interclass correlation coefficient (ICC) and ordinal measures using Cohen’s Kappa (CBCT zone categories).

The non-parametric Mann–Whitney (M-W) rank sum test was used to distinguish differences in tooth positions (rotation, M-D inclination or B-P inclination) of the lateral incisors or first premolars between the impacted canine sites and the control sites. The chi-square test or Fisher’s exact test was used to further differentiate in which directions the teeth were misaligned.

The Mann–Whitney rank sum test and the *t*-test for independent samples were used to compare buccally and palatally impacted canines in relation to malpositions (rotation, M-D inclination or B-P inclination) of the lateral incisors and first premolars.

The presence of a mucosal bulge was compared based on the shortest distance between the cortical bone and the impacted tooth or the dental follicle of the impacted tooth using an independent samples *t*-test.

The correlation between the malpositions of the lateral incisors or premolars, their maximum follicle thickness and the CBCT distances between the tooth and the impacted canine was analyzed using Pearson’s correlation.

Kruskal–Wallis (K-W) one-way analysis of variance was performed to analyze the associations between the vertical or horizontal zone of the canine with malpositions (rotation, M-D inclination or B-P inclination) of the lateral incisors or first premolars.

The relationships for some variables are graphically represented by boxplots and scatterplots. In boxplots, the centre box represents the range between the 25th and 75th percentiles, the solid line represents the median and the whiskers represent the 5th and 95th percentiles. Circles and dots show distribution of the sample. For statistically significant relationships, a line with a slope coefficient is drawn, shaded red areas represent the 95% confidence intervals. Unless otherwise stated, the values are given as mean ± standard deviation (SD). An asterisk (*) indicates a *p*-value ≤0.05.

## 4. Results

The study included 62 participants (35 females, 27 males, average age 14.7 years), who were evenly divided into two groups: 31 participants in the study group and 31 participants in the control group. In the study, 26 palatally impacted canines (PIC), 5 buccally impacted canines (BIC), 31 contralateral non-impacted canines and 62 non-impacted canines of the control group were examined. Age, gender and skeletal maturity according to CVM stage in the impacted canine group and control group are reported in Table 1. Measurements were made by the same researcher and showed excellent reliability ICC values between 0.96 and 1 and Cohen Kappa values near 1.

### 4.1. Position of the Contralateral Canine

**Figure 5:**
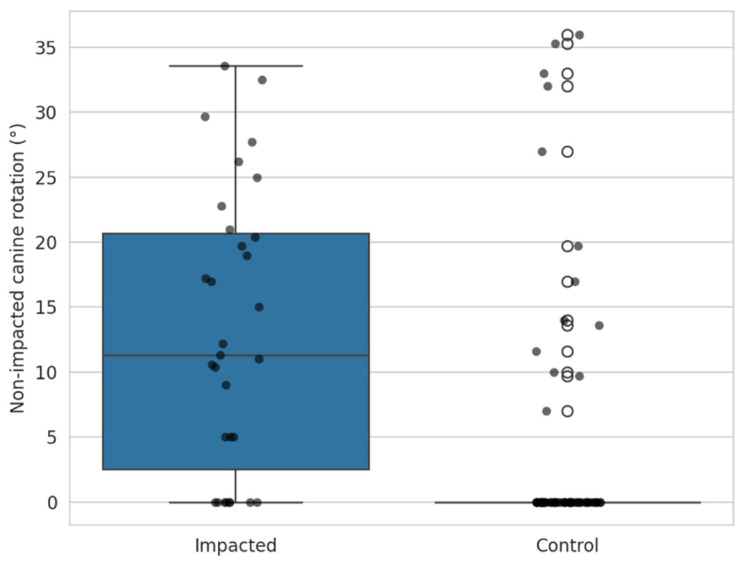
Rotation of the contralateral non-impacted canine in the impacted group compared to the canines in the control group.

The rotation of the contralateral canine in the impacted group was compared with the canine of the control group using the Mann–Whitney test. The patients in the impacted group had a significantly higher rotation of the contralateral canines (*p* < 0.001). Figure 5.

### 4.2. Position of the Lateral Incisors and the 1st Premolars in Relation to the Impacted Canine

**Figure 6:**
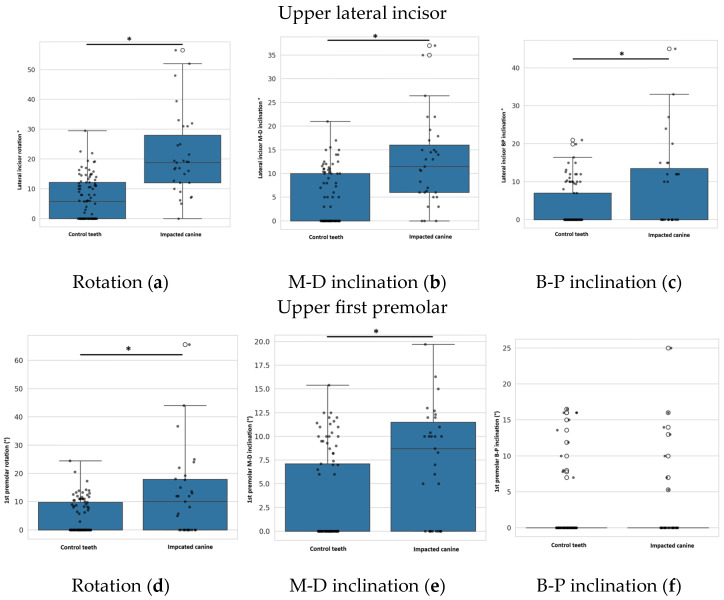
Rotation (**a**), M-D inclination (**b**) and B-P inclination (**c**) of lateral incisor adjacent to impacted canine compared to control teeth. Rotation (**d**), M-D inclination (**e**) and B-P inclination (**f**) of 1st premolar adjacent to impacted canine compared to control teeth. An asterisk (*) indicates a statistically significant difference with *p*-value ≤ 0.05.

Compared to the control non-impacted site, lateral incisors adjacent to the site of impacted canine was significantly more rotated (*p* < 0.001, M-W test), had a significantly greater M-D inclination (*p* < 0.001, M-W test) and a significantly greater B-P inclination (*p* = 0.013, M-W test). Figure 6a–c. In the lateral incisors, there were no differences between the impacted canines and the control teeth in terms of the type of rotation (mesiobuccal vs. mesiodistal), M-D inclination (mesial vs. distal) or B-P inclination (buccal vs. palatal).

Lateral incisors adjacent to the impacted canines showed significantly greater rotation, mesiodistal inclination, and buccopalatal inclination compared with controls, though the type of deviation did not differ.

The first premolars were significantly more rotated on the impacted side compared to the non-impacted sides (*p* = 0.003, M-W test), had significantly higher M-D inclinations (*p* < 0.001, M-W test), but did not show higher B-P inclinations (*p* = 0.156, M-W test). Figure 6d–f. In the first premolars, no differences in the type of rotation (mesiobuccal vs. mesiodistal), M-D inclinations (mesial vs. distal) or B-P inclinations (buccal vs. palatal) were observed between the impacted canine and the control teeth.

First premolars on the impacted side also had greater rotation and mesiodistal inclination, but no significant change in buccopalatal inclination.

### 4.3. Influence of the Position of the Impacted Canine (Buccal or Palatal) on the Position of the Lateral Incisors and the 1st Premolars

Based on the CBCT images, the impacted canines were classified into two groups according to their anatomical position. The malpositions of the lateral incisors and first premolars were documented using 3D study models.

We found that buccally impacted maxillary canines significantly influenced the position of the lateral incisors. Lateral incisors adjacent to buccally impacted canines had significantly higher rotation values *p* = 0.03 (*t*-test), significantly higher M-D inclinations *p* = 0.008 (M-W test) and higher B-P inclinations *p* < 0.001 (M-W test). Table 2

Position of the upper 1*st* premolar was equally affected in both in buccally as well as palatally impacted adjacent maxillary canine (*p* > 0.05). Table 2

### 4.4. Mucosal Bulge

The mucosal bulge over the impacted maxillary canines was recorded on the 3D study models. The sites of impacted canines were categorized into visible and non-visible mucosal bulges. The shortest distances between the impacted canine and the outer cortical bone margin and the shortest distances between the follicle of the impacted canine and the outer cortical bone margin were measured on CBCT images.

**Figure 7:**
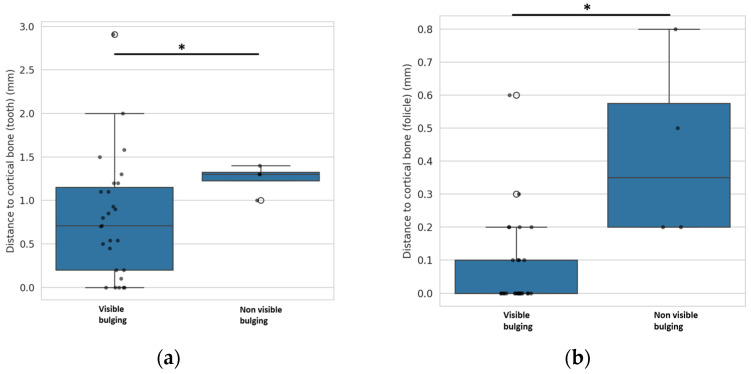
Visibility of mucosal bulge in relation to CBCT measured distance between outer cortical bone margin and impacted canine (**a**) or impacted canine dental follicle (**b**). An asterisk (*) indicates a statistically significant difference with *p*-value ≤ 0.05.

The distance between the impacted canine and the outer cortical bone margin was significantly greater (at 1.125 ± 0.173 mm) in the canines where no mucosal bulge was present than in the canines where a mucosal bulge was visible (0.789 ± 0.695 mm) (*p* = 0.008, *t*-test). Figure 7a. Based on the data obtained, we found that the threshold value at which a mucosal bulge is no longer present is at a distance between the canine and the outer bone margin of more than 1.2 mm.

The distance between the follicle of the impacted canine and the outer cortical bone margin was significantly greater in canines where no mucosal bulge was present: 0.425 ± 0.287 mm compared to 0.078 ± 0.0.137 mm where a mucosal bulge was visible (*p* < 0.001, *t*-test). Figure 7b.

Based on the data obtained, we were able to determine that the threshold value at which a mucosal bulge is no longer present is at a distance between the follicle of the canine and the outer bone margin of more than 0.2 mm.

CBCT analysis showed that visible mucosal bulges over impacted canines are linked to shorter distances between the canine or its follicle and the outer cortical bone. Bulges disappeared when the canine–bone margin exceeded 1.2 mm or when the follicle–bone margin exceeded 0.2 mm.

### 4.5. Association Between the Malpositions of the Lateral Incisor and 1st Premolar Adjacent to the Impacted Canine Site

The correlation between the values of rotation, M-D and B-P inclination was calculated for the lateral incisors adjacent to impacted maxillary canines. The analysis showed that all malpositions were significantly correlated (Pearson correlation), the correlation can be interpreted as moderate to strong (Table 3).

The association between the premolar values of rotation, M-D and B-P inclination was analyzed using the Pearson correlation. A low correlation was observed between all measures (Table 3).

### 4.6. Influence of the Horizontal or Vertical Zone of the Impacted Canine on the Distance to the Lateral Incisor or Premolar

Using the one-way analysis of variance model, it was shown that the influence of the anatomical zone of the impacted canine in its vertical and horizontal anatomical position according to the CBCT image had no influence on the distance to the lateral incisor measured with CBCT or on its malpositions measured on the 3-D study models (*p* > 0.05, one-way ANOVA).

The vertical or horizontal anatomical position of the impacted canine according to the CBCT image was not significantly associated with the CBCT-measured distance to the first premolar or the 3D measured values of the rotation, M-D or B-P inclination of the first premolar (*p* > 0.05, one-way ANOVA).

Analysis showed that the vertical and horizontal anatomical position of impacted canines on CBCT had no significant effect on their distance to adjacent teeth or on the positional changes in the lateral incisors and first premolars, indicating these malpositions occur independently of canine location.

### 4.7. Correlation Between Maximum Follicle Thickness, Distance Between Canine and Neighbouring Teeth and Malpositions of Neighbouring Teeth

**Figure 8:**
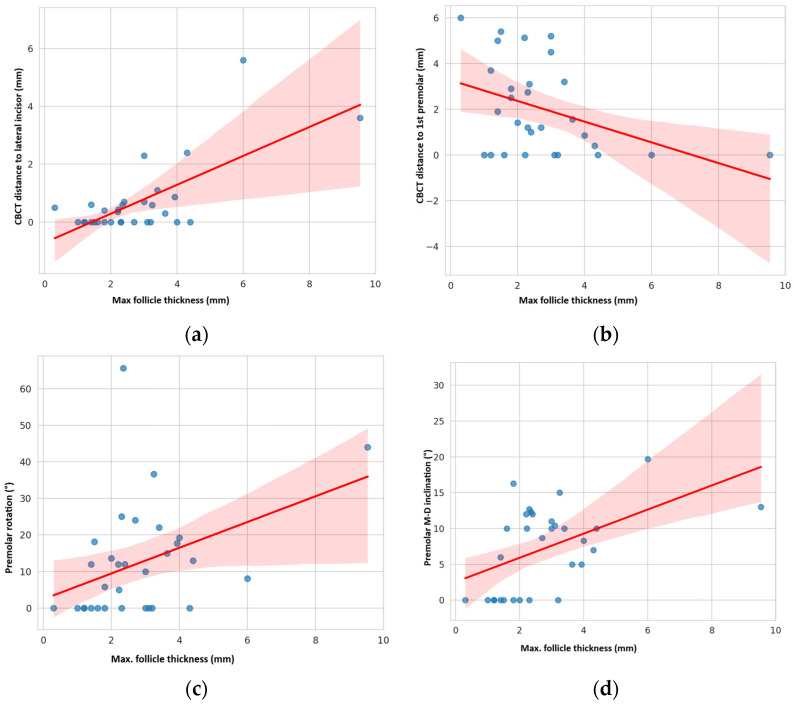
Correlation between maximal impacted canine follicle thickness and distance to lateral incisor (**a**) or distance to 1st premolar (**b**). Maximal follicle thickness was positively correlated with 1st premolar rotation (**c**) and its M-D inclination (**d**).

A strong positive correlation was found between the maximum follicle thickness and the shortest distance between the impacted canine and the lateral incisor measured by CBCT: R = 0.702, *p* < 0.001 (Pearson correlation). Figure 8a. Maximum follicle thickness did not correlate with the improper position of lateral incisor (*p* > 0.05).

Conversely, a moderate negative correlation was found between the maximum follicle thickness and the distance between the impacted canine and the first premolar: R = −0.403, *p* = 0.030 (Pearson correlation). Figure 8b. The maximum follicle thickness correlated moderately with the greater rotation of the first premolar (R = 0.403, *p* = 0.024) and its M-D inclination (R = 0.498, *p* = 0.004, Pearson correlation). Figure 8c–d.

Follicle thickness showed a strong positive correlation with the canine–lateral incisor distance but no link to incisor malposition. In contrast, greater follicle thickness correlated negatively with the canine–premolar distance and moderately with increased premolar rotation and mesio-distal inclination.

## 5. Discussion

Based on the results of this clinical study, patients with unilaterally impacted canines showed a significantly greater rotation of the contralateral canine than patients in the group without impacted canines. The lateral incisor adjacent to the impacted canine showed significantly greater rotation and higher M-D and B-P inclination, while the first premolars showed significantly greater rotation and higher M-D inclination. The lateral incisors were significantly more misaligned when they were adjacent to buccally impacted canines. Conversely, the position of the first premolars did not differ depending on the anatomical position of the first premolar. A visible mucosal bulge above the impacted canine indicates a smaller distance between the canine and its follicle to the external cortical bone. A thicker follicle of the impacted canine correlated with a greater distance of the impacted canine to the L-I and a shorter distance to the F-P. A thicker follicle increased the rotation and M-D inclination of the F-P.

Analysis of the rotation of the canines revealed that the canines on the contralateral side were statistically significantly more rotated than in the control group, suggesting that positional abnormalities of the canines are present in both impacted canines and contralateral canines in patients with unilateral canine impaction. An unerupted permanent canine together with a rotated contralateral permanent canine could be an indication of a possible unilateral canine impaction, and this combination of clinical signs could signal the need for further radiographic investigation.

The results of the study show an increase in positional discrepancies, especially in terms of rotation and M-D angulation of the LI and FP near impacted maxillary canines compared to regions without canine impaction. Similar results have been reported in other studies suggesting that due to the rare positioning of the impacted canine in the premolar region, its presence does not frequently affect the buccopalatal inclination of the first premolar [21,28].

When comparing buccally and palatally impacted canines, significant differences in position can be observed, particularly in the lateral incisors (LIs), especially with buccal impaction. Results indicate that buccally impacted canines lead to a greater rotational displacement, mesiodistal (M-D) angulation and buccopalatal (B-P) inclination of adjacent LIs compared to palatally impacted canines. No statistically significant difference was found in the position of the first premolar (FP) between the two impaction types.

Our findings align with Dekel et al. [21], showing more severe rotation of LIs in both palatally (17.1°) and buccally (18°) impacted canine groups compared to controls. The greater LI rotation in our buccally impacted group might be due to its smaller size and the direct relationship between LI rotation and canine position [21,29]. While Dekel et al. [21] found no affected M-D angulation in buccally impacted LI, our study observed higher M-D angulation differences in this group, potentially due to our smaller sample size and the severity of canine impaction and its relation to nearby roots. Jacobs [30] suggests that proclined and distally tipped LIs can indicate buccal canine impaction.

Both, our study and by Dekel et al. [21] found comparable root displacement of LIs: palatally impacted canines displaced LI roots buccally (mean 5°), while buccally impacted canines displaced them palatally (mean 15.8°). Regarding FPs, both studies show significant rotation of FP crowns in impacted canine groups (6–7°) compared to controls. While our study found greater FP M-D angulation, Dekel et al. [21] also noted that closer proximity of the impacted canine to the FP increases FP M-D angulation. Otherwise, no other significant FP positional differences were observed in either study.

Analyzing the influence of the vertical and horizontal zones on the position of the LI and FP did not yield statistically significant results. This can be attributed to the limited sample size within the individual subgroups, which led to a low-test power. Nevertheless, the results are consistent with other studies. In this study, the results of analyzing the influence of the horizontal zone on the position of the LI showed that the median value of its rotation was highest in horizontal zone 1, reaching 25°. This result is comparable with studies by R. J. Olive and Denkel et al. [21,29], who reported that the degree of mesiolabial rotation of the LI is most pronounced in cases where the canines are positioned closer to the midline. The highest values of M-D angulation of the LI were observed in horizontal zones 1 and 3, which can be explained by the mechanical influence of the impacted canine, which displaces the LI laterally. Liuk et al. [23] reported that the neighbouring impacted canine was positioned on a higher vertical plane relative to the occlusal plane when the crown of the LI had a lower inclination in the sagittal or coronal plane (the LI crown tilted more palatally or mesially). However, our study could not confirm these results.

This study also aimed to investigate how the size of the dental follicle and the positional relationship between the adjacent teeth and the dental follicle of the impacted canine influenced the relative position of the adjacent LI and FP. The maximum follicle thickness showed a moderate correlation with the rotation and mesiodistal angulation of the first premolar, but no significant correlation was found with the malposition of the lateral incisor. Several studies have found that impacted maxillary teeth tend to have enlarged follicles, which can undergo pathological changes [31,32,33]. Enlarged dental follicles are often a sign of impaired eruption potential of the tooth [34,35].

Ericson and Bjerklin [36], suggested that the displacement of adjacent roots is most likely caused by the eruptive force of the maxillary canine and not by the dental follicle itself. However, they also acknowledged that it remains unclear whether an enlarged dental follicle during eruption of the canine increases the risk of displacement of adjacent incisor roots. In the study by M. Lam et al. [37], no statistically significant difference was found in the relative position of the adjacent lateral incisor and first premolar in relation to the follicular volume of the impacted canine compared to the control side. However, their results suggested that the direct physical contact between the canine crown and the adjacent root may play a more important role in root displacement. Our results are consistent with these observations. The thickness of the follicle did not have a major influence on the lateral incisor; on the contrary, thicker follicles influenced the position of the FP. It is currently unclear whether the follicle may play a role in causing impaction [35] or whether the impaction itself stimulates follicle growth. Since the evidence is mostly limited to the 3rd molars, observation of follicles in impacted canines could be a good subject for further studies.

A clinical examination can provide valuable information about an impacted canine even before radiographs are taken. The first step in prevention or interception is to recognize a possible impaction of the canines. The dentist must also look for irregularities in the position of the neighbouring teeth and the contralateral permanent canine, which may indicate impaction of the canines and their position. It is important that the diagnosis of possible canine impaction is made early, as relatively simple interceptive procedures can be performed at the right time during tooth development, such as extraction of the primary canines, space maintenance in the affected area with a transpalatal arch or space gain through expansion or extraoral traction [38,39], and could reduce the risk of impaction and avoid the need for lengthy, costly and complex (and with a higher potential for side effects) combined ortho-surgical treatment [22,40,41]. The combination of the clinical signs described could serve as an indicator of a possible impaction if radiological assessment is not (yet) possible or available.

Visual inspection and digital palpation are the first, simplest and least expensive methods to determine that a canine is impacted and to determine the position of the impacted tooth. In one study, 70% of impacted teeth could be palpated and it could be determined whether the impaction of the canine was palatal or buccal [29].

However, in some cases, the bony canine protrusion may be mistaken for the impacted tooth, or the impacted canine may not even be recognized through visual inspection or digital palpation due to its ectopic position, which is outside the palpable cortical surface.

Based on the data obtained, we determined that the threshold value at which a mucosal bulge is no longer present is a distance between the canine and the outer bone margin of more than 1.2 mm, while the threshold value for the distance between the follicle of the canine and the outer bone margin is more than 0.2 mm. The visual bulge thus shows that the impacted tooth is relatively close to the outer surface of the alveolar bone. To our knowledge, these thresholds have not been specifically measured or reported in any previous study.

A limitation of the study is the very small number of buccally impacted canines, which limits the reliability of subgroup analyses. Additionally, the distribution of impacted canines across the different vertical and horizontal CBCT zones is uneven. The small buccal impaction sample, combined with this uneven zonal distribution, reduces the statistical power to compare characteristics across subgroups. Since palatal impactions are much more common in the general population, a study specifically designed to compare a larger sample of buccally impacted canines with palatally impacted control teeth would provide a more robust framework for evaluating their features. Further validation with a larger sample is necessary to confirm the observed differences, which did not reach statistical significance. In future research, it would be useful to analyze the dental follicles that fall within the extremely large ranges of size measurements. Perhaps a threshold for follicle size needs to be exceeded to have a significant effect on the position of neighbouring teeth. Future research should also investigate the chronological development of malocclusion of the lateral incisors and first premolars in cases with impacted maxillary canines.

## 6. Conclusions

This study revealed differences in the rotation, mesiodistal angulation and buccopalatal inclination of the lateral incisor adjacent to impacted canines compared to non-impacted controls.

The first premolar adjacent to the impacted canine showed increased rotation and mesiodistal angulation, while buccopalatal inclination remained unchanged.

When comparing the discrepancies between buccally and palatally impacted canines, positional discrepancies were only observed in the lateral incisors.

The canines on the contralateral side exhibited significantly greater rotation than those in the control group.

A visible bulge above the impacted canine indicates a smaller distance between the canine and its follicle and the outer cortical bone.

The thickness of the impacted canine follicle can influence the position of the first premolar, while it has no influence on the lateral incisor.

## Figures and Tables

**Table 1:** There were no differences between the participants in impacted canine group in gender, age or skeletal maturity according to CVM stage.

	Impacted Canine	Control Group	
Frequency (N)	31	31	
Age (median in years)	14.34	14.28	*p* = 0.598 (M-W test)
Gender	17 f, 14 m	18 f, 13 m	*p* = 1 (Chi-square test)
Skeletal maturity (median CVM)	5	4	*p* = 0.621 (M-W test)

**Table 2:** (mal)positions of lateral incisors and 1st premolars based on anatomical location of canine impaction.

	Buccal	Palatal	*p*-Value (test)
Lateral incisor Rotation° (mean ± SD)	33.12 (±21.25)	18.73 (±11.01)	* 0.03 (*t*-test)
Lateral incisor M-D inclination° median (25–75%)	22 (15.45–31.7)	9.45 (5–14.7)	* 0.008 (M-W test)
Lateral incisor B-P inclination° median (25–75%)	24 (16–36)	0 (0–12)	* <0.001 (M-W test)
1st premolar rotation° median (25–75%)	0 (0–10.55)	12 (0–19.9)	0.166 (M-W test)
1st premolar M-D Inclination° Median (25–75%)	10 (0–15.05)	8.5 (0–12)	0.891 (M-W test)
1st premolar B-P Inclination° median (25–75%)	0 (0–8)	0 (0–1.33)	1.0 (M-W test)

* Signifies a statistically significant value of <0.05.

**Table 3:** Correlation between malpositions of lateral incisors or premolars.

Correlation Between	Correlation Coefficient (R)	*p*-Value
Lateral incisor Rotation-M-D inclination	0.71	<0.001
Lateral incisor Rotation-B-P inclination	0.59	<0.001
Lateral incisor M-D-B-P inclination	0.53	<0.001
1st premolar Rotation-M-D inclination	0.32	<0.001
1st premolar Rotation-B-P inclination	0.39	<0.001
1st premolar M-D-B-P inclination	0.38	<0.001

## Data Availability

Data is available on request due to restrictions. Part of the data contains sensitive personal information and is therefore protected by the national and EU (GDPR) laws. However, data that could not compromise the privacy of research participants may be available from the corresponding author (AG) upon reasonable request.
